# Approaching the diagnosis of growth-restricted neonates: a cohort study

**DOI:** 10.1186/1471-2393-10-6

**Published:** 2010-02-01

**Authors:** Popi Sifianou

**Affiliations:** 1 Department of Neonatology, General & Maternity Hospital "Elena Venizelou", 2 Elenas Venizelou Sq., 11521 Athens, Greece

## Abstract

**Background:**

The consequences of *in utero *growth restriction have been attracting scholarly attention for the past two decades. Nevertheless, the diagnosis of growth-restricted neonates is as yet an unresolved issue. Aim of this study is the evaluation of the performance of simple, common indicators of nutritional status, which are used in the identification of growth-restricted neonates.

**Methods:**

In a cohort of 418 consecutively born term and near term neonates, four widely used anthropometric indices of body proportionality and subcutaneous fat accretion were applied, singly and in combination, as diagnostic markers for the detection of growth-restricted babies. The concordance of the indices was assessed in terms of positive and negative percent agreement and of Cohen's kappa.

**Results:**

The agreement between the anthropometric indices was overall poor with a highest positive percent agreement of 62.5% and a lowest of 27.9% and the κ ranging between 0.19 and 0.58. Moreover, 6% to 32% of babies having abnormal values in just one index were apparently well-grown and the median birth weight centile of babies having abnormal values of either of two indices was found to be as high as the 46^th ^centile for gestational age (95%CI 35.5 to 60.4 and 29.8 to 63.9, respectively). On the contrary, the combination of anthropometric indices appeared to have better distinguishing properties among apparently and not apparently well-grown babies. The median birth weight centile of babies having abnormal values in two (or more) indices was the 11^th ^centile for gestational age (95%CI 6.3 to 16.3).

**Conclusions:**

Clinical assessment and anthropometric indices in combination can define a reference standard with better performance compared to the same indices used in isolation. This approach offers an easy-to-use tool for bedside diagnosis of *in utero *growth restriction.

## Background

'The diagnosis of impaired fetal growth in newborn infants continues to depend largely on two major parameters: birth weight and gestational age'. This is the introductory statement in a paper by Miller and Hassanein [1] on the diagnosis of impaired growth in newborns, which aimed at documenting the insufficiency of using birth weight to uncover fetal growth disturbances. Almost forty years later, a neonatal test that produces a definitive diagnosis of *in utero *growth-restricted babies is not yet available. Consequently, small for gestational age babies are taken as *in utero *growth-restricted (IUGR), despite increased awareness that the two terms are not synonymous [2].

From a theoretical perspective growth-restricted neonates could be detected through reduced prenatal growth [2]. Nevertheless, in addition to the numerous potential errors involved in biometric measures [3] there is no consistently superior parameter reflecting fetal growth accurately and the most commonly used fetal biometric parameters were found to correlate poorly with size at birth [4]. Doppler velocitometry and components of the biophysical profile, in combination, are definitively superior regarding diagnostic accuracy [5], even though these approaches have not been standardized [6].

Pediatricians are called to identify IUGR babies promptly and accurately, so as treat appropriately even those who have had no medical care prenatally, i.e. all IUGR babies irrespective of the level of prenatal care. Therefore, an easy-to-use tool for bedside diagnosis of growth-restricted neonates is desirable.

## Methods

### Subjects

All consecutive singleton babies, delivered after 35 weeks of gestational age (GA), at General & Maternity Hospital "Elena Venizelou", during four randomly selected weekly periods, were prospectively studied.

### Data collection

Babies were evaluated and measured between 12 and 24 hours of life, except for birth weight (BW) which was recorded at birth. The evaluation included assessment of nutritional status and of GA, using the Expanded New Ballard Score [7]. The former was based on the Clinical Assessment of Nutritional Status (CANS) scoring method [8], which evaluates subcutaneous fat accretion at eight body locations and features of the hair. In the present study, this last criterion was replaced by one evaluating the skin, under the following formulation: Skin well hydrated, vernix caseosa possibly present especially in body folds (4 points); rather dry skin, peeling over palm and soles, vernix caseosa absent even in babies of 37 to 38 weeks gestation (3 points); skin overall dry, desquamating on the extremities (2 points); skin peeling off in large flakes, parchmentlike skin (1 point) [9]. GA was calculated in completed weeks from the last menstrual period and compared with that derived from babies' clinical assessment. If in disagreement for over 2 weeks the clinical score was recorded.

Included in the measurements were: a) the birth length (BL), b) the largest occipitofrontal circumference (HC), c) the chest circumference (CC) at the level just below the nipples and d) the mid-arm circumference (MAC) at the midpoint between acromion and olecranon of the right arm placed next to the chest with the palm facing the thigh. Circumferences were measured to the nearest 0.1 cm with a plastic tape measure of 0.9 cm width. Birth length was measured by Rollameter (Harlow Printed Ltd., UK) to the nearest 0.1 cm. All measurements were taken in triplicate and the mean was recorded. At the end of the data collection ponderal index (PI), i.e. weight in g/(length in cm)^3 ^× 100, and the ratio MAC/HC were calculated. Abnormal values of the anthropometric indices and of BW were defined as values ≤ 10^th ^percentile for GA. *Per *definition a CANS score ≥ 27 describes apparently well-grown babies [8]. Pregnancy and delivery history were obtained by reviewing the medical records and by interviewing the mother. During the interview, mothers' written informed consent for the inclusion of their babies in the study was also obtained. The study protocol was approved by the Ethical Committee of the General & Maternity Hospital "Elena Venizelou".

### Data analysis

Statistical analysis was performed using MedCalc for Windows, version 10.4 (MedCalc Software, Mariakerke, Belgium). Anthropometric measurements were expressed as percentiles. Mann-Whitney test for independent samples was used to determine differences between anthropometric indices of individual groups of babies and chi-square test for categorical variables. p < 0.05 was considered statistically significant. The agreement of diagnostic markers was estimated using the Cohen's kappa for chance-corrected agreement as well as the positive and negative percentage agreement [10,11]. Positive agreement of two indices was calculated as the number of cases having abnormal values in both indices divided by the sum of cases with abnormal values in each index. Negative agreement was calculated in the same way taking into account the normal values of the indices [12].

## Results

The study included 418 consecutively born, singleton neonates between 35 and 41 weeks GA (208 boys/210 girls). No statistically significant sex difference was detected in MAC (p = 0.08), PI (p = 0.07), CC (p = 0.13) and MAC/HC (p = 0.32). GA estimation was based on clinical evaluation in 15 cases: in 3 cases with unavailable last menstrual period data and in another12 due to the disagreement between clinical assessment and maternal dates; in 9 of the last 12 cases a history of irregular menses was present.

### Agreement of the anthropometric indices

The agreement of the anthropometric indices was studied in terms of positive (p_pos_) and of negative (p_neg_) percent agreement, as well as of chance-corrected agreement, i.e. Cohen's kappa. As shown in Table 1 the agreement beyond chance between the anthropometric indices was fair, with κ values ranging between 0.19 and 0.33. The only exception was the agreement between MAC and MAC/HC, which appeared stronger probably owing to their common component. The κ was 0.58 and the p_pos _62.5%.

**Table 1:** Agreement of the anthropometric indices

	MAC to PI	MAC to CC	MAC to MAC/HC	PI to CC	PI to MAC/HC	CC to MAC/HC
*p*_pos_	27.9%	40.0%	62.5%	29.3%	29.2%	32.6%

*p*_neg_	91.0%	92.7%	95.1%	92.3%	91.6%	92.2%

***κ***	0.19	0.33	0.58	0.21	0.21	0.25
**SE**	0.09	0.08	0.07	0.09	0.09	0.09
**95%CI**	0.01 to 0.39	0.16 to 0.49	0.44 to 0.71	0.03 to 0.40	0.03 to 0.39	0.07 to 0.43

Kappa statistics, positive (p_pos_) and negative (*p*_neg_) percent agreement between the anthropometric indices dichotomized at the 10^th ^centile for gestational age. MAC, mid-arm circumference; PI, ponderal index; CC, chest circumference; HC, head circumference

**Figure 1:**
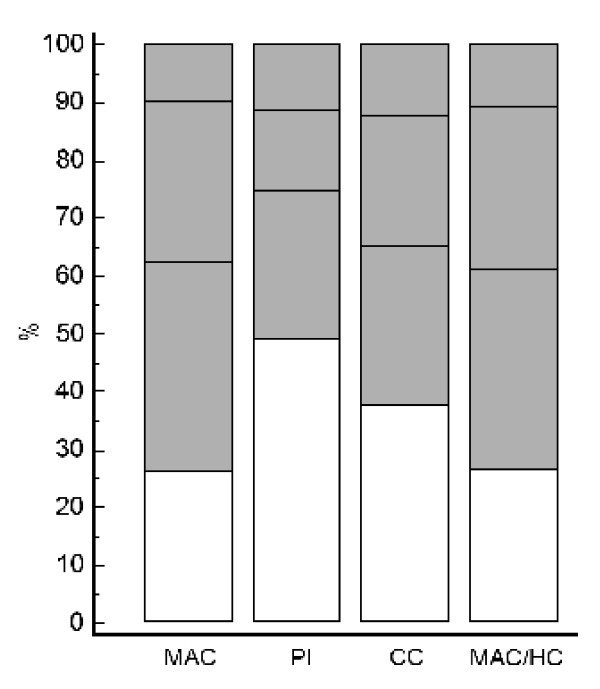
**Proportion of babies with abnormal values in one or more anthropometric indices**. Columns stand for all cases with abnormal values (≤ 10^th ^centile for gestational age) in the individual indices. Unfilled areas of columns represent cases with abnormal values of only the indicated index. The three shaded areas of each column represent cases with abnormal values of both the indicated index and of any 1, 2 or 3 additional indices, from bottom to top, respectively. MAC, mid-arm circumference; PI, ponderal index; CC, chest circumference; HC, head circumference

Given the low level of agreement, all individual cases with abnormal values in one anthropometric index were subsequently examined for co-occurrence of abnormal values in all three remaining indices. For instance, all cases having PI ≤ 10^th ^centile were tested for values of MAC ≤ 10^th ^centile and those cases with abnormal PI, but normal MAC values, were tested for abnormal CC values and so forth. The percentage of cases with abnormal values of PI and of at least one more index was 51% (22 cases out of a total of 43 cases with abnormal values of PI in the study population). Hence, almost half of the cases with abnormal PI values agreed with at least one more of the three remaining indices (and the other half with none). This percentage was 74% (37/50) for cases with MAC ≤ 10^th ^centile, 62.5% (25/40) for cases with CC ≤ 10^th ^and almost 74% (34/46) for cases with MAC/HC ≤ 10^th ^centile. These results are illustrated in Figure 1. Unfilled areas in the columns represent the proportion of cases with abnormal values of the indicated index but normal values of all three remaining indices.

### Misclassification of babies as IUGR by using anthropometric indices singly

BW centiles and CANS scores of babies having abnormal values in a single index were compared with those of babies having abnormal values of this same index and at least one more. Mann-Whitney test for independent samples was used to assess the statistical significance of the differences. The results are collectively presented in Table 2. Cases with abnormal values in each of the four indices were divided into two sub-groups on the basis of the presence or absence of abnormal values of other indices. The sub-group S includes cases having abnormal values in a single index and the sub-group C those having abnormal values in a combination of indices. Babies with abnormal values of MAC were found to have comparable CANS scores irrespective of the presence or absence of abnormal values of the other three indices; median CANS scores of both the S and C sub-groups of babies having MAC ≤ 10^th ^centile were 22 (p = 0.32). However, CANS scores were significantly different between babies having abnormal values of only PI or CC or MAC/HC (S sub-groups) and babies having abnormal values of more than one of the indices (C sub-groups). For instance, median CANS score of cases having only PI ≤ 10^th ^centile (S sub-group) was 27 *versus *22 of the C sub-group (p < 0.0001). Moreover, taking into account that a CANS score ≥ 27 describes, *per *definition, babies with apparently normal subcutaneous fat mass, it was evident that apparently well-grown babies had abnormal values in a single index, e.g., 14 out of 21 babies with abnormal values of PI but normal values of MAC, CC and MAC/HC. On the contrary, none of the cases with abnormal values of PI and of at least one more index (C sub-group) was apparently well-grown; median CANS score was 22 in the latter group versus 27 in the former. Consequently, if PI, CC or MAC/HC were used as single indicators of growth restriction, a relatively high proportion of babies designated as IUGR would be well-grown babies; 32.6%, 17.5% and 10.9%, respectively.

**Table 2:** Comparison of babies having abnormal values of one or more indices

		**CANS**					**BW centiles**		
			
		***n***	***Median***	***95%CI***	***p***	***Score ≥ 27 (no/total)***	***Median***	***95% CI***	***p***
	
**MAC≤ **	**S**	13	22	21.9 to 25.0	.32	0/13	12.7	5.8 to 18.3	.44
**10^th ^centile**	**C**	37	22	21.0 to 24.0		3/37	9.5	4.6 to 15.7	
	
**PI≤ **	**S**	21	27	24.0 to 27.2	<.0001	14/21	46.5	35.5 to 60.4	<.0001
**10^th ^centile**	**C**	22	22	20.0 to 24.0		0/22	10.8	4.1 to 21.8	
	
**CC≤ **	**S**	15	26	21.0 to 27.0	.01	5/15	10.4	5.3 to 22.3	.03
**10^th ^centile**	**C**	25	22	20.4 to 23.8		2/25	5.5	2.1 to 11.2	
	
**MAC/HC≤ **	**S**	12	25	23.6 to 27.0	.0002	3/12	45.2	29.8 to 63.9	<.0001
**10^th ^centile**	**C**	34	22	21.0 to 24.0		2/34	12.3	5.5 to 21.1	

All babies with abnormal values (≤ 10^th ^centile for gestational age) of the individual indices are classified into two groups: The first, symbolized by S, includes cases with abnormal values in a *single index *(the one indicated in the left hand column) and the second, symbolized by C, includes cases with abnormal values in a *combination of indices *(including the one indicated in the left hand column). The p values refer to the S and C groups of the individual indices. CANS, Clinical Assessment of Nutritional Status Score; BW, birth weight; MAC, mid-arm circumference; PI, ponderal index; CC, chest circumference; HC, head circumference

A similar picture emerged when BW centiles were taken into consideration (Table 2). The median BW centile of babies having abnormal only PI or only MAC/HC values (S sub-groups) was the 46^th ^centile, an inappropriately high median BW centile for supposedly *in utero *growth-restricted babies. BW centiles were significantly lower in the groups of babies who had more than one abnormal value in anthropometric index (C sub-groups) compared to the groups of babies who had only one abnormal value (S sub-groups). Again, in addition to CANS scores, no statistically significant differences in BW centiles were found in the two groups of babies with abnormal MAC values.

As a group, the median BW centile of babies having abnormal values in only one anthropometric index was 26.8 (95%CI 17.8 to 37.6) versus 11.4 (95%CI 6.3 to 16.3) in babies having abnormal values in at least two indices (p < 0.0001). The corresponding median CANS scores were 25 (95%CI 24.0 to 26.5) and 22 (95%CI 21 to 24), respectively (p < 0.0001).

### Categorization of study babies on the basis of abnormal values of indices

Overall 47 babies (out of 418 studied) were found to have abnormal values in two or more of the four anthropometric indices. Of those, 24 babies were appropriate and 23 small for GA. In the total population, 328 babies were appropriate and 47 small for GA. Thus, the prevalence of babies having abnormal values in at least two indices was 7.4% and 48.9% among appropriate and small for GA, respectively. The profile of babies having abnormal values in none, one and two or more of the anthropometric indices, as well as some maternal characteristics are summarized in additional file 1, Table S3: Profile of babies with and without abnormal values in the four anthropometric indices.

## Discussion

The high rate of morbidities in growth-restricted neonates has been well documented. Moreover, accumulated evidence over the last two decades converges on an increasing risk of metabolic syndrome among individuals who have experienced growth restriction during fetal life [13]. For both these reasons the distinction between growth-restricted and non-restricted babies is of paramount importance.

Irrespective of cause, fetuses with inadequate nutrition will not deposit fat as long as their basic metabolic needs are not met. Conversely, a baby with abundant subcutaneous fat cannot have suffered from *in utero *malnutrition. On the basis of this principle, the evaluation of fat deposits is an appropriate means for the distinction between IUGR and non-IUGR neonates. To this end, anthropometry has been carried out for years. Indeed, numerous studies dealing with short or long term consequences of *in utero *growth restriction consider their subjects as growth-restricted if the ratios BW to BL (principally PI), MAC, the ratio MAC to HC and less frequently CC are lower than a given threshold value. Rarely is the distinction between IUGR and non-IUGR babies based on clinical signs at birth suggestive of fetal malnutrition, in an atypical [14] or in a structured form, like CANS score [8]. Both the anthropometric indices and the clinical evaluation of nutritional status have been proven more sensitive predictors of early neonatal morbidities, ascribed to *in utero *growth restriction, as compared to BW [15-18].

Despite their interchangeable use in the relevant studies, the above diagnostic markers of *in utero *growth restriction perform differently, as evidenced in the present study. Only 28% of babies with MAC ≤ 10^th ^centile had also PI at or below this level. Since high accuracy entails high agreement [19], the relatively low level of agreement between the anthropometric indices could be ascribed to their low diagnostic accuracy in the identification of IUGR babies. This assumption is supported by the relatively high proportion of babies, found in the present study, who had abnormal values of individual indices, despite their being apparently well-nourished, e.g. 32.6% of cases having abnormal PI values. The low diagnostic performance of PI is in agreement with other studies [17,20].

Whenever a reference standard is not available, the optimal method that has been suggested for the distinction between diseased and non-diseased individuals is the combination of several imperfect diagnostic tests [21]; in the broad sense of the term 'test' [22]. Depending on the availability of a nearly perfect test, on the diagnostic performance of individual tests, on their interdependence, etc. several methods and rules for combining tests have been developed [23]. Moreover, the combination of several diagnostic tests appears to be a reasonable approach for a highly complex and multi-factorial process, like intrauterine growth. *In utero *growth restriction is not a uniform condition with respect to its severity and duration, the underlying pathogenesis and the developmental stage of the fetus at the time of its occurrence. Therefore, a single anthropometric index or any other test cannot suffice to detect all babies with impaired *in utero *growth accurately. In the present study, the combination of anthropometric indices proved to have better performance in the diagnosis of not apparently well-grown babies over the isolated use of the same indices.

A diagnostic test should have the potential to be implemented in clinical practice. Moreover, IUGR babies should be identified immediately after birth, so as to receive the appropriate care promptly. Contrary to more sophisticated imaging techniques, which are expensive and impractical to use in clinical settings, anthropometry is not only a relatively simple, but also a reliable tool for bedside quantification of body composition and proportions. A noticeable limitation of all the anthropometric indices mentioned is their dependence on GA. Subsequently, any inaccurate estimation of GA will impact on the accuracy of the identification of IUGR neonates (which, however, also holds for BW). By contrast, this problem does not pertain to CANS score, which is unrelated to GA. This scoring method helps the clinician get insights into babies' nutritional status, by focusing on those body areas where subcutaneous fat should have been accumulated during *in utero *life, and eventually quantify his evaluation. Its major drawback is its subjective nature, like all other scoring methods used in the evaluation of neonates. The method could be used as a screening or confirmatory test.

All in all, the combined over the isolated use of anthropometric measurements appears to offer a better approach in the identification of growth-restricted babies. In every term or near term baby with clinical signs of wasting (e.g., absence of chin fat-folds, skin easily grasped and lifted in fold, visible or prominent ribs, reduced gluteal fat) MAC and CC can be measured at bedside easily [24]. In addition, PI and the ratio MAC/HC can be calculated using measures included in neonatal records. Babies with abnormal values in more than one anthropometric index can be managed as growth-restricted. Abnormal values in more than one index in apparently well-grown babies may necessitate a re-evaluation of GA. Undoubtedly, further research is needed, using a greater range of confirmatory information. Search and evaluation of alternative indices or other simple indicators of growth restriction might also contribute to a more accurate identification of IUGR babies.

## Conclusions

Research evidence of many decades points to *in utero *growth restriction as a leading cause of early neonatal morbidity. It is highly likely that at least part of it (e.g. hypoglycemia, especially in appropriate for GA babies) escape our attention due to the lack of a precise diagnostic tool. To this end, the idea of a combined reference standard, as the one proposed above, can improve our capacity to identify and manage growth-restricted babies appropriately.

## Competing interests

The author declares that she has no competing interests.

## Pre-publication history

The pre-publication history for this paper can be accessed here:

http://www.biomedcentral.com/1471-2393/10/6/prepub

## Supplementary Material

Additional file 1 **Profile of babies with and without abnormal values in the four anthropometric indices**. Characteristics of babies (and their mothers) allocated into three groups: babies with abnormal values (≤ 10^th ^centile for gestational age) in none, in one, and in two or more anthropometric indices. The p values refer to the two preceding groups. GA, gestational age; B, boys; G, girls; BW, birth weight; BL, birth length; HC, head circumference; MAC, mid-arm circumference; PI, ponderal index; CC, chest circumference; HC, head circumference; CANS, Clinical Assessment of Nutritional Status Click here for file
