# Supplementary material for: Approaching the diagnosis of growth-restricted neonates: a cohort study
Source: BMC Pregnancy Childbirth. 2010 Feb 1;10:6. doi: 10.1186/1471-2393-10-6 (PMC2830965; doi:10.1186/1471-2393-10-6)
Supplement: Additional file 1 — Profile of babies with and without abnormal values in the four anthropometric indices. Characteristics of babies (and their mothers) allocated into three groups: babies with abnormal values (≤ 10th centile for gestational age) in none, in one, and in two or more anthropometric indices. The p values refer to the two preceding groups. GA, gestational age; B, boys; G, girls; BW, birth weight; BL, birth length; HC, head circumference; MAC, mid-arm circumference; PI, ponderal index; CC, chest circumference; HC, head circumference; CANS, Clinical Assessment of Nutritional Status [file 1471-2393-10-6-S1.PDF]

	4 indices > 10th centile			1 index ≤ 10th centile				2 or more indices ≤ 10th centile			
	n	***Median***	***95%CI***	***n***	***Median***	***95%CI***	***p***	***n***	***Median***	***95%CI***	***p***
GA (wks)	310	38	38-39	61	38	38-39	0.99	47	38	38-39	0.99
Sex (B/G)	310	151/159		61	34/27		0.31	47	23/24		0.55
BW (g)	310	3350	3293-3405	61	3040	2840-3142	<0.0001	47	2800	2654-2875	<0.0001
BL (cm)	310	49.8	49.6-50.1	61	49.5	48.5-50.4	0.15	47	48.2	47.7-49.3	0.01
HC (cm)	310	34.4	34.3-34.5	61	34.1	33.3-34.6	0.01	47	33.6	33.3-34.2	0.09
MAC(cm)	310	9.7	9.6-9.8	61	9.0	8.8-9.2	<0.0001	47	8.5	8.3-8.7	<0.0001
PI (g/cm^3)	310	2.69	2.66-2.70	61	2.49	2.42-2.57	<0.0001	47	2.44	2.35-2.53	0.06
CC (cm)	310	32.7	32.5-32.9	61	31.2	30.7-31.7	<0.0001	47	30.5	29.5-30.8	0.0006
MAC/HC	310	0.28	0.28-0.28	61	0.26	0.26-0.27	<0.0001	47	0.25	0.25-0.26	<0.0001
CANS score	310	27	27-27	61	25	24.0-26.5	<0.0001	47	22	21-24	<0.0001
Maternal age (yrs)	310	29	28-30	61	28	26-30	0.80	47	28	26-30	0.95
Pre-pregnancy weight (kg)	233	61	59.1-63.0	47	60	55.0-64.5	0.54	34	55.5	52.0-62.9	0.14
Weight gain (kg)	230	14	13.4-15.0	48	13.2	10.0-15.0	0.09	33	12	10.3-14.0	0.76
Smoking (yes/no)	250	32/218		50	9/41		0.45	34	3/31		0.39
